# P-1926. Once Is Enough, Two Is Too Much: Unraveling Predictors of COVID-19 Readmission among Previously Hospitalized Patients

**DOI:** 10.1093/ofid/ofae631.2086

**Published:** 2025-01-29

**Authors:** Marta Colaneri, Marta Canuti, Ginevra Torrigiani, Lucia Dall’Olio, Chiara Bobbio, Sante Leandro Baldi, Alessandro Nobili, Massimo Puoti, Giulia Marchetti, Nicola Latronico, Pierluigi Plebani, Mario Raviglione, Andrea Gori, Maria Luisa Ojeda Fernandez, Marta Baviera, Mauro Tettamanti, Olivia Leoni, Ida Fortino, Alessandra Bandera

**Affiliations:** Department of Biomedical and Clinical Sciences, University of Milan, Milan, Italy, Milano, Lombardia, Italy; Department of Veterinary and Animal Sciences, University of Copenhagen, Copenhagen, Denmark, Copenaghen, Hovedstaden, Denmark; Department of Statistics and Quantitative Methods, Università degli Studi di Milano-Bicocca & Laboratory of Cardiovascular Prevention, Istituto di Ricerche Farmacologiche Mario Negri IRCCS, Milan, Italy, Milano, Lombardia, Italy; Centre for Multidisciplinary Research in Health Science (MACH), University of Milan, Milan, Italy, Milano, Lombardia, Italy; Department of Medical-Surgical and Transplant Pathophysiology, Fondazione IRCCS Ca’ Granda Ospedale Maggiore Policlinico, Milan, Italy, Milano, Lombardia, Italy; Centre for Multidisciplinary Research in Health Science (MACH), University of Milan, Milan, Italy, Milano, Lombardia, Italy; Department of Health Policy, Istituto di Ricerche Farmacologiche Mario Negri IRCCS, Milan, Italy, Milano, Lombardia, Italy; Department of Infectious Diseases, ASST Grande Ospedale Metropolitano Niguarda, Milan, Italy, Milano, Lombardia, Italy; Clinic of Infectious Diseases, Department of Health Sciences, ASST Santi Paolo e Carlo, University of Milan, Milan, Italy, Milano, Lombardia, Italy; Department of Emergency, ASST Spedali Civili University Hospital, Piazzale Ospedali Civili, 1, 25123 Brescia, Italy, Brescia, Lombardia, Italy; Department of Electronics, Information and Bioengineering, Politecnico di Milano, Milan, Italy, Milano, Lombardia, Italy; Centre for Multidisciplinary Research in Health Science (MACH), University of Milan, Milan, Italy, Milano, Lombardia, Italy; Infectious Diseases and Immunopathology, Department of Clinical Sciences, Università di Milano, Luigi Sacco Hospital, Milan, Italy, Milano, Lombardia, Italy; Laboratory of Cardiovascular Prevention, Istituto di Ricerche Farmacologiche Mario Negri IRCCS, Milan, Italy, Milano, Lombardia, Italy; Laboratory of Cardiovascular Prevention, Istituto di Ricerche Farmacologiche Mario Negri IRCCS, Milan, Italy, Milano, Lombardia, Italy; Laboratory of Geriatric Epidemiology, Istituto di Ricerche Farmacologiche Mario Negri IRCCS, Milan, Italy, Milano, Lombardia, Italy; Welfare General Directorate, Lombardy Region, Milan, Italy, Milano, Lombardia, Italy; Welfare General Directorate, Lombardy Region, Milan, Italy, Milano, Lombardia, Italy; Department of Medical-Surgical and Transplant Pathophysiology, Fondazione IRCCS Ca’ Granda Ospedale Maggiore Policlinico, Milan, Italy, Milano, Lombardia, Italy

## Abstract

**Background:**

While risk factors for COVID-19 hospitalization are well characterized, only a few studies investigated those associated with hospital re-admission after SARS-CoV-2 re-infections, since most are focused-on re-admission because of all-causes. Our aim was to identify co-morbidities and other risk factors associated with a second COVID-19 hospitalization.

**Figure:**
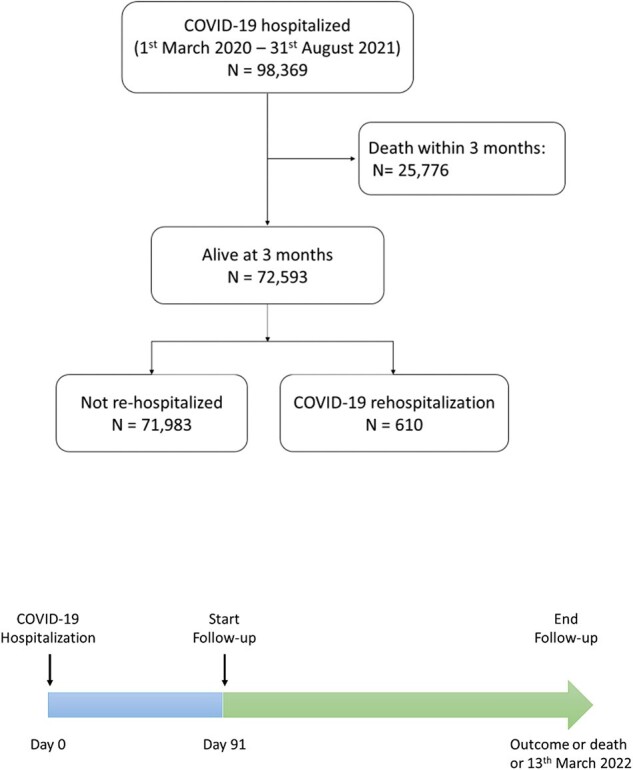

Study flowchart and timeline

**Methods:**

Administrative database from the Lombardy Welfare Directorate was used to detect patients hospitalized for COVID-19 for a first time between 1^st^ Feb 2020 to 31^st^ Aug 2021. Information on demographic variables, hospital records and drug prescriptions were collected. The population comprised the re-hospitalized individuals, who were readmitted for COVID-19 at least 3 months after their initial hospital admission, likely because of a different SARS-CoV-2 infection and the non-re-hospitalized subjects. Follow-up observation lasted until the re-hospitalization, death or the end of study period. Adjusted hazard ratio (CI, 95%) was performed to assess the association between risk factors and re-hospitalization with a competing risk analysis.

**Figure:**
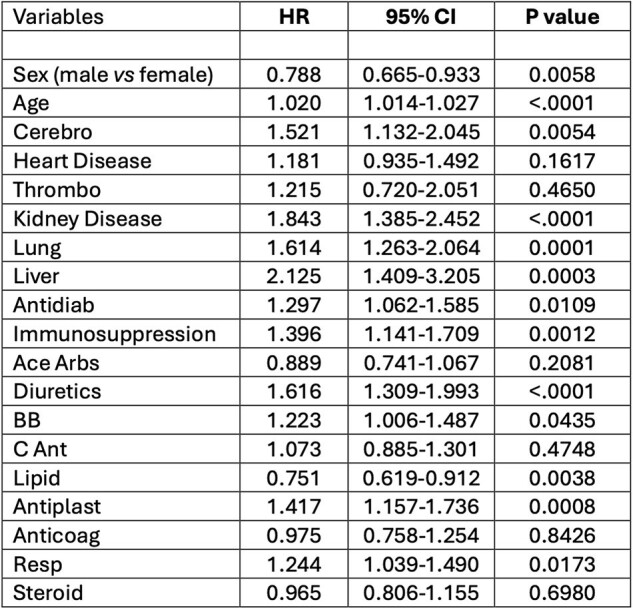

Multivariable analysis with competing risk, where death after 3 months is the competing risk for re-hospitalization due to COVID-19.

**Results:**

From a total of 98,369 patients hospitalized for COVID-19, 25,776 (26.2%) died within three months and 610 (0.6%) were re-hospitalized for a likely new SARS-CoV-2 infection (Figure 1). Re-hospitalized patients were mostly male (61.3%) and were 72.2 years on average.

The strongest risks for re-hospitalization in patients with a new episode of COVID-19 were kidney and liver diseases. However, a significant association for this risk was also observed for age, males, immune suppression, cerebro-vascular and cardio-vascular diseases, lung disease, diabetes, and some medications (Table 1).

**Conclusion:**

We demonstrated that vulnerability due to multimorbidity and chronic conditions, such as kidney and liver disfunction, may diminish the ability to withstand physiological challenges, elevating the risk of re-hospitalization. The fact that the elderly, immune suppressed individuals, or those with chronic conditions may survive an initial hospitalization does not safeguard them from further admissions. We emphasized the importance of targeted interventions and heightened clinical vigilance for these high-risk patient populations.

**Disclosures:**

All Authors: No reported disclosures

